# Complete mitochondrial genome of the yellow-legged Asian hornet, *Vespa velutina nigrithorax* (Hymenoptera: Vespidae)

**DOI:** 10.1080/23802359.2017.1285211

**Published:** 2017-02-06

**Authors:** Jong Seok Kim, Jun Seong Jeong, Iksoo Kim

**Affiliations:** College of Agriculture & Life Sciences, Chonnam National University, Gwangju, Republic of Korea

**Keywords:** Mitochondrial genome, *Vespa velutina nigrithorax*, invasive hornet

## Abstract

The yellow-legged Asian hornet, *Vespa velutina nigrithorax*, which originated from Asia, has invaded several countries, including South Korea. In Korea, *V. velutina nigrithorax* predation on honeybees is one of the most serious factors threatening apiculture. We sequenced the complete mitochondrial genome (mitogenome) of *V. velutina* to better understand the mitogenomic characteristics of this species. The 16,475 bp mitogenome of *V. velutina* consists of a typical set of genes, with an arrangement identical to that of congeneric species. *Vespa velutina* possesses the shortest A + T-rich region (132 bp) among congeneric *Vespa*, and this is also the shortest in the superfamily Vespoidea. Phylogenetic analysis using the 13 protein-coding genes of Vespoidea species indicated that each family forms strongly supported monophyletic groups (Bayesian posterior probability =1; ML, 100%). Moreover, *V. velutina* and *V. bicolor* form strongly supported sister groups (Bayesian posterior probability =1; ML, 100%).

Originally distributed from northern India to the Indochinese Peninsula, Taiwan, and Indonesia (Rortais et al. 2010) the yellow-legged Asian hornet, *Vespa velutina nigrithorax*, has invaded Europe and also South Korea (Haxaire et al. 2006; Kim et al. 2006; Castro & Pagola-Carte 2010; Bruneau 2011; Villemant et al. 2011). First observed in Korea in 2003 (Kim et al. 2006), the species has now spread to nearly all regions of South Korea, the sole exception being the remote island of Jeju. This species is one of the major apicultural pests, and is also a pest in urban areas. During summer, the economic losses caused by this devastating bee-hawking hornet are so severe that beekeeping becomes almost unviable.

In this study, we sequenced the complete mitochondrial genome (mitogenome) of *V. velutina* to better understand the mitogenomic characteristics of this species and its phylogenetic relationships within Vespoidea. In 2016, one adult *V. velutina* was captured at Juam-myeon, in Jeollanamdo Province in Korea (35°04′16.1″N, 127°13′38.8′′E) using a commercial hornet trap. This voucher specimen was deposited in Chonnam National University, Gwangju, Korea, under the accession no. CNU6596.

Using total DNA as a template, three long overlapping fragments (COI–ND4, ND4-lrRNA, and lrRNA–COI) were amplified, and subsequently, 32 short overlapping fragments were amplified using the three long fragments as templates. The primers used in the present study for amplifying the long- and short-fragments were designed from available *Vespa* mitogenomes (Chen et al. 2016; Wei et al. 2016).

The *V. velutina* mitogenome is 16,475 bp in size and includes the typical sets of genes (2 rRNAs, 22 tRNAs, and 13 protein-coding genes [PCGs]) and a major non-coding A + T-rich region (GenBank accession number KY091645). At 16,476 bp, the mitogenome of *V. velutina* is larger than other *Vespa* mitogenomes that have been completely sequenced to date (15,779 bp in *V. ducalis*, Kim et al., In Press; 15,902 bp in *V. mandarinia*, Chen et al. 2016). The gene arrangement in the *V. velutina* mitogenome is identical to that of other *Vespa* species, but it differs substantially from the ancestral insect order found in the majority of insects (Boore 1999).

The A/T content among genes and regions varies markedly in the *V. velutina* mitogenome: 92.4% in the A + T-rich region, 86.0% in tRNAs, 85.6% in *lrRNA*, 84.4% in *srRNA*, and 79.7% in PCGs. All *V. velutina* PCGs begin with the typical ATN codon (six with ATG, five with ATT, one with ATC, and one with ATA). Twelve of the 13 PCGs end with TAA, whereas COIII ends with a single T. The incomplete termination codon results in a complete TAA stop codon via post-translational modifications occurring during the mRNA maturation process (Ojala et al. 1981).

We performed phylogenetic analysis using the 13 PCGs of 14 mitogenome sequences from Vespoidea, including that of *V. velutina,* with the inclusion of one species each from Ichneumonidae and Evaniidae belonging to Vespoidea as outgroups (Wei et al. 2009, 2010). Both Bayesian inference (BI) and maximum-likelihood (ML) methods (Stamatakis, 2006; Ronquist et al. 2012) were performed using the GTR + GAMMA + I model in CIPRES Portal v. 3.1 (Miller et al. 2010). Using these two analyses, identical topologies were obtained, with both Formicidae and Vespidae forming strongly supported monophyletic groups (Figure 1; Bayesian posterior probability = 1; ML, 100%). Within *Vespa*, the sister relationships between *V. ducalis* and *V. bicolor* and between *V. mandarinia* and *V. ducalis* were strongly supported (BPP = 1; ML, 97–100%). Previous phylogenetic analysis of *Vespa* based on combined analyses of morphological (45 characters) and molecular (four mt genes and two nuclear genes) data also support the close relationships between *V. ducalis* and *V. bicolor* and between *V. mandarinia* and *V. ducalis* (Perrard et al. 2013).

**Figure 1.**
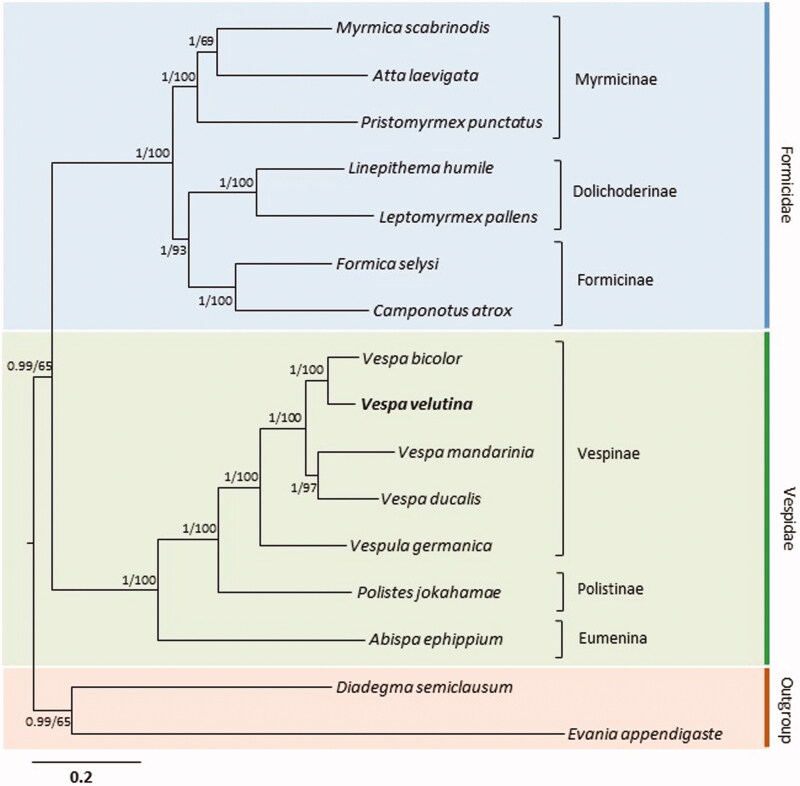
Phylogeny of Vespoidea. Bayesian Inference (BI) and maximum-likelihood (ML) methods produced the same topology based on 13 concatenated protein-coding genes. The numbers at each node specify percentage Bayesian posterior probabilities generated by BI analysis (first value) and bootstrap percentages of 1000 pseudo-replicates generated by ML analysis (second value). The scale bar indicates the number of substitutions per site. One species from the Ichneumonidae (*Diadegma semiclausum*) and one species from the Evaniidae (*Evania appendigaste*) were utilized as outgroups. GenBank accession numbers are as follows: *V. ducalis*, KX950825; *V. mandarinia*, KR059904; *V. bicolor*, KJ735511; *Vespula germanica*, KR703583; *Polistes jokahamae*, KR052468; *Abispa ephippium*, EU302588; *Atta laevigata*, KC346251; *Myrmica scabrinodis*, LN607806; *Pristomyrmex punctatus*, AB556946; *Camponotus atrox*, KT159775; *Formica selysi*, KP670862; *Linepithema humile*, KX146468; *Leptomyrmex pallens*, KC160533; *D. semiclausum*, EU871947; and *E. appendigaste*, FJ593187.
